# A potent risk model for predicting new-onset acute coronary syndrome in patients with type 2 diabetes mellitus in Northwest China

**DOI:** 10.1007/s00592-020-01484-x

**Published:** 2020-02-01

**Authors:** Jun Lyu, Zhiying Li, Huiyi Wei, Dandan Liu, Xiaoxian Chi, Da-Wei Gong, Qingbin Zhao

**Affiliations:** 1 Clinical Research Center, The First Affiliated Hospital of Xi’an Jiaotong University, Xi’an, 710061 Shaanxi China; 2 Department of Geratology, The First Affiliated Hospital of Xi’an Jiaotong University, Xi’an, 710061 Shaanxi China; 3 The Second Affiliated Middle School of Xi’an Jiaotong University, Xi’an, 710061 Shaanxi China; 4 Division of Endocrinology, Diabetes and Nutrition, Department of Medicine, University of Maryland School of Medicine, Baltimore, 21201 USA

**Keywords:** Cardiovascular disease, Type 2 diabetes mellitus, Risk predictive model, Northwest China

## Abstract

**Aims:**

Type 2 diabetes mellitus (T2DM) is now very prevalent in China. Due to the lower rate of controlled diabetes in China compared to that in developed countries, there is a higher incidence of serious cardiovascular complications, especially acute coronary syndrome (ACS). The aim of this study was to establish a potent risk predictive model in the economically disadvantaged northwest region of China, which could predict the probability of new-onset ACS in patients with T2DM.

**Methods:**

Of 456 patients with T2DM admitted to the First Affiliated Hospital of Xi’an Jiaotong University from January 2018 to January 2019 and included in this study, 270 had no ACS, while 186 had newly diagnosed ACS. Overall, 32 demographic characteristics and serum biomarkers of the study patients were analysed. The least absolute shrinkage and selection operator regression was used to select variables, while the multivariate logistic regression was used to establish the predictive model that was presented using a nomogram. The area under the receiver operating characteristics curve (AUC) was used to evaluate the discriminatory capacity of the model. A calibration plot and Hosmer–Lemeshow test were used for the calibration of the predictive model, while the decision curve analysis (DCA) was used to evaluate its clinical validity.

**Results:**

After random sampling, 319 and 137 T2DM patients were included in the training and validation sets, respectively. The predictive model included age, body mass index, diabetes duration, systolic blood pressure (SBP), diastolic blood pressure (DBP), low-density lipoprotein cholesterol, serum uric acid, lipoprotein(a), hypertension history and alcohol drinking status as predictors. The AUC of the predictive model and that of the internal validation set was 0.830 [95% confidence interval (CI) 0.786–0.874] and 0.827 (95% CI 0.756–0.899), respectively. The predictive model showed very good fitting degree, and DCA demonstrated a clinically effective predictive model.

**Conclusions:**

A potent risk predictive model was established, which is of great value for the secondary prevention of diabetes. Weight loss, lowering of SBP and blood uric acid levels and appropriate control for DBP may significantly reduce the risk of new-onset ACS in T2DM patients in Northwest China.

## Background

At present, diabetes has become an epidemic in developing countries, posing a serious threat to people’s health with increasing medical burden. The prevalence of type 2 diabetes mellitus (T2DM) in Chinese adults (2018) was 12.75%. Diabetes is an independent risk factor for cardiovascular disease (CVD). Compared with non-diabetic population, the risk of CVD in diabetic patients is 2–4 times higher [1–3]. Having diabetes is often associated with important risk factors of CVD such as hypertension and dyslipidaemia [4]. Clinical evidence shows that strict blood glucose control offers a limited effect in reducing the risk of CVD and related deaths in patients with T2DM, especially those with a longer course of disease, the older age, those with previous CVD, or with multiple cardiovascular risk factors [5]. However, comprehensive control of multiple risk factors can markedly decrease the risk of CVD and deaths in patients with diabetes [6]. Currently, in Chinese T2DM patients, the incidence of cardiovascular risk factors is high, but the control rate is low. In patients with T2DM who are treated in the outpatient clinics, the comprehensive compliance rate of blood glucose, blood pressure and blood lipid controlling was only 5.6% [4].

Acute coronary syndrome (ACS) is the most serious clinical phenotype of CVD. Although there are many treatments for ACS, the mortality, recurrent myocardial infarction and repeated hospitalization of ACS patients remain high. Most of the hospitalized ACS patients have dysglycaemia [7]. In clinical practice in the low-income Northwest China, diabetes is very commonly found in ACS patients in the intensive coronary care unit. In this setting, diabetes is often complicated with coronary multi-vessel diseases, often requiring multiple percutaneous coronary interventions (PCIs), with a high incidence of repeated hospitalization, heart failure and poor prognosis.

Due to the high incidence of T2DM in low-income areas in China, there are high mortality and heavy medical burden of ACS among diabetic patients. Thus, this study aimed to establish a potent risk predictive model, which can provide early prediction of the risk of new-onset ACS in T2DM patients, thereby reducing the adverse cardiovascular outcomes in such patients in the region.

## Methods

### Study design and setting

This study collected data from 456 T2DM patients who were admitted to the First Affiliated Hospital of Xi’an Jiaotong University from January 2018 to January 2019. The ethics committee of the medical centre approved the study (Reference Number: XJTU1AF2019LSK-064).

Patient demographic characteristics, serum biomarkers and coronary angiography data were collected by trained researchers at the study centre. All data were then submitted electronically to a central department for verification. All experiments were approved by the First Affiliated Hospital of Xi’an Jiaotong University, and all methods were carried out in accordance with international guidelines and regulations. A written informed consent was obtained from all participants.

### Study participants

This study randomly selected 456 T2DM patients hospitalized in the medical centre from January 2018 to January 2019. Of these, 186 had both T2DM and ACS and were newly diagnosed either with ST-segment elevation myocardial infarction or non-ST-segment elevation myocardial infarction, or unstable angina, according to the recommended criteria by the Joint European Society of Cardiology and the American College of Cardiology Committee [8]. The remaining 270 patients had T2DM without ACS. The diagnosis of T2DM was based on the World Health Organization and American Diabetes Association (ADA) criteria as follows: fasting plasma glucose concentration of 7.0 mmol/L (126 mg/dL) or higher, or 2-h post-glucose load venous plasma glucose of 11.1 mmol/L (200 mg/dL) or higher, confirmed on two occasions. The exclusion of type 1 diabetes mellitus was based on clinical distinction by the respective attending physicians, including young age of onset, positive islet autoantibody and history of insulin-dependent glycaemic control. Furthermore, gestational diabetes and other types of diabetes were also excluded.

All ACS patients underwent coronary angiography during the hospitalization, performed by at least two interventional doctors in the Department of Cardiology of the medical centre.

### Candidate predictors

The collected demographic variables included age, sex, body mass index (BMI), smoking status, alcohol drinking status, hypertension history, T2DM duration, family history of coronary heart disease (CHD), systolic blood pressure (SBP) and diastolic blood pressure (DBP). BMI was calculated as weight (kg)/height (m^2^), which were measured at admission.

Serum biomarkers included blood urea nitrogen, serum creatinine, cystatin C, serum uric acid (SUA), estimated glomerular filtration rate, total cholesterol, triglycerides, low-density lipoprotein cholesterol (LDL-C), high-density lipoprotein cholesterol (HDL-C), lipoprotein(a) [Lp(a)], apolipoprotein A (ApoA), apolipoprotein B (ApoB), apolipoprotein E (ApoE), platelet count, platelet distribution width, mean platelet volume, platelet–larger cell ratio, plateletcrit, haemoglobin A1c (HbAlc), fasting blood glucose, D-dimer and gamma-glutamyl transpeptidase.

### Statistical analysis

The continuous variables with normal distribution were expressed as mean ± standard deviation, while the categorical variables were expressed as percentages. To establish and validate the predictive model, 70% of the study patients were randomly selected as the training set and the other 30% as the validation set. The least absolute shrinkage and selection operator (LASSO) regression was used to select variables into the predictive model. By applying multivariate logistic regression, we established a predictive model. The risk predictive model of new-onset ACS in T2DM patients was presented using a nomogram. We evaluated the predictive model from three aspects, namely the discriminatory capacity, the calibration ability and the clinical effectiveness. The discriminatory capacity was evaluated by the area under the receiver operating characteristics (ROC) curve (AUC). The calibration ability was evaluated by a calibration plot and Hosmer–Lemeshow test. The clinical effectiveness was evaluated by the decision curve analysis (DCA).

All tests were two-tailed, and a *P* value of < 0.05 was considered statistically significant. All statistical analyses were performed using R software (version 3.5.1, Vienna, Austria).

## Results

### Participant characteristics

Following random sampling, 319 and 137 T2DM patients were included in the training and validation sets, respectively. The demographic and clinicobiochemical characteristics of the participants are listed in Table 1.

**Table 1:** Participant characteristics

Variables	Training set (*n* = 319)	Validation set (n = 137)	*P* value
Status, *n*(%)			0.153
0 = without ACS	182 (57.1)	88 (64.2)	
1 = with ACS	137 (42.9)	49 (35.8)	
Sex, *n*(%)			0.64
1 = male	219 (68.7)	91 (66.4)	
2 = female	100 (31.3)	46 (33.6)	
Age, years	56.7 ± 10.2	57.2 ± 9.9	0.591
T2DM duration, years	8.6 ± 5.7	9.3 ± 6.7	0.244
Family history of CHD, *n*(%)			0.141
1 = yes	38 (11.9)	10 (7.3)	
2 = no	281 (88.1)	127 (92.7)	
Hypertension, *n*(%)			0.610
1 = yes	155 (48.6)	63 (46.0)	
2 = no	164 (51.4)	74 (54.0)	
Drinking, *n*(%)			0.013
1 = yes	37 (11.6)	28 (20.4)	
2 = no	282 (88.4)	109 (79.6)	
Smoking, *n*(%)			0.879
1 = yes	121 (37.9)	53 (38.7)	
2 = no	198 (62.1)	84 (61.3)	
Body mass index, kg/m^2^	25.8 ± 3.7	25.2 ± 3.9	0.131
Systolic blood pressure, mmHg	132.7 ± 18.1	130.7 ± 15.7	0.262
Diastolic blood pressure, mmHg	78.4 ± 10.2	76.8 ± 9.7	0.124
Haemoglobin Alc, %	8.1 ± 1.8	8.1 ± 1.8	0.879
Fasting blood glucose, mmol/L	8.1 ± 2.9	7.7 ± 2.6	0.195
Total cholesterol, mmol/L	4.0 ± 0.9	4.1 ± 1.0	0.271
Triglyceride, mmol/L	1.9 ± 1.6	1.8 ± 1.4	0.623
High-density lipoprotein cholesterol, mmol/L	1.0 ± 0.3	1.0 ± 0.3	0.646
Low-density lipoprotein cholesterol, mmol/L	2.4 ± 0.8	2.5 ± 0.8	0.175
Apolipoprotein A, g/L	1.1 ± 0.2	1.2 ± 0.2	0.342
Apolipoprotein B, g/L	0.8 ± 0.2	0.8 ± 0.2	0.341
Apolipoprotein E, mg/L	35.8 ± 16.7	35.6 ± 16.5	0.920
Lipoprotein(a), mg/L	154.9 ± 162.5	153.8 ± 169.9	0.950
Estimated glomerular filtration rate, ml/min/1.73 m^2^	123.6 ± 34.1	124.9 ± 30.4	0.688
Blood urea nitrogen, mmol/L	5.8 ± 1.8	5.7 ± 1.6	0.591
Serum creatinine, mmol/L	62.1 ± 22.1	59.5 ± 15.4	0.210
Cystatin C, mg/L	0.8 ± 0.3	0.8 ± 0.2	0.309
Serum uric acid, μmol/L	334.2 ± 80.1	333.4 ± 86.1	0.920
D-dimer, mg/L	0.5 ± 0.5	0.4 ± 0.2	0.419
Gamma-glutamyl transpeptidase, U/L	31.1 ± 24.0	34.2 ± 41.2	0.320
Platelet count, × 109/L	193.9 ± 55.6	189.5 ± 52.7	0.436
Platelet distribution width, fL	15.5 ± 3.0	15.5 ± 3.1	0.917
Mean platelet volume, fL	11.7 ± 1.3	11.7 ± 1.3	0.842
Platelet–larger cell ratio, %	38.3 ± 10.3	38.8 ± 10.1	0.658
Plateletcrit, %	0.2 ± 0.1	0.2 ± 0.1	0.285

ACS, acute coronary syndrome; T2DM, type 2 diabetes mellitus; CHD, coronary heart disease

### Independent risk factors in the training set

Multivariate logistic regression analysis demonstrated that age, BMI, diabetes mellitus duration, SBP, DBP, LDL-C, SUA, Lp(a), hypertension history and alcohol drinking status were independent risk factors for new-onset ACS in T2DM patients (Table 2).

**Table 2:** Multivariate logistic regression analysis (training set)

Variables	OR	95% CI	*P* value
Age	1.067	1.033–1.103	< 0.001
Body mass index	1.139	1.055–1.235	0.001
Diabetes duration	0.943	0.893–0.992	0.027
Systolic blood pressure	1.031	1.010–1.053	0.005
Diastolic blood pressure	0.940	0.903–0.969	< 0.001
Low-density lipoprotein cholesterol	0.547	0.371–0.792	0.002
Serum uric acid	1.008	1.004–1.012	< 0.001
Lipoprotein(a)	1.002	1.000–1.004	0.028
Hypertension			
1 = yes	Reference		
2 = no	0.417	0.232–0.741	0.003
Drinking			
1 = yes	Reference		
2 = no	1.497	0.621–3.864	0.383

OR, odds ratio; CI, confidence interval

### Predictive model construction

**Fig. 1:**
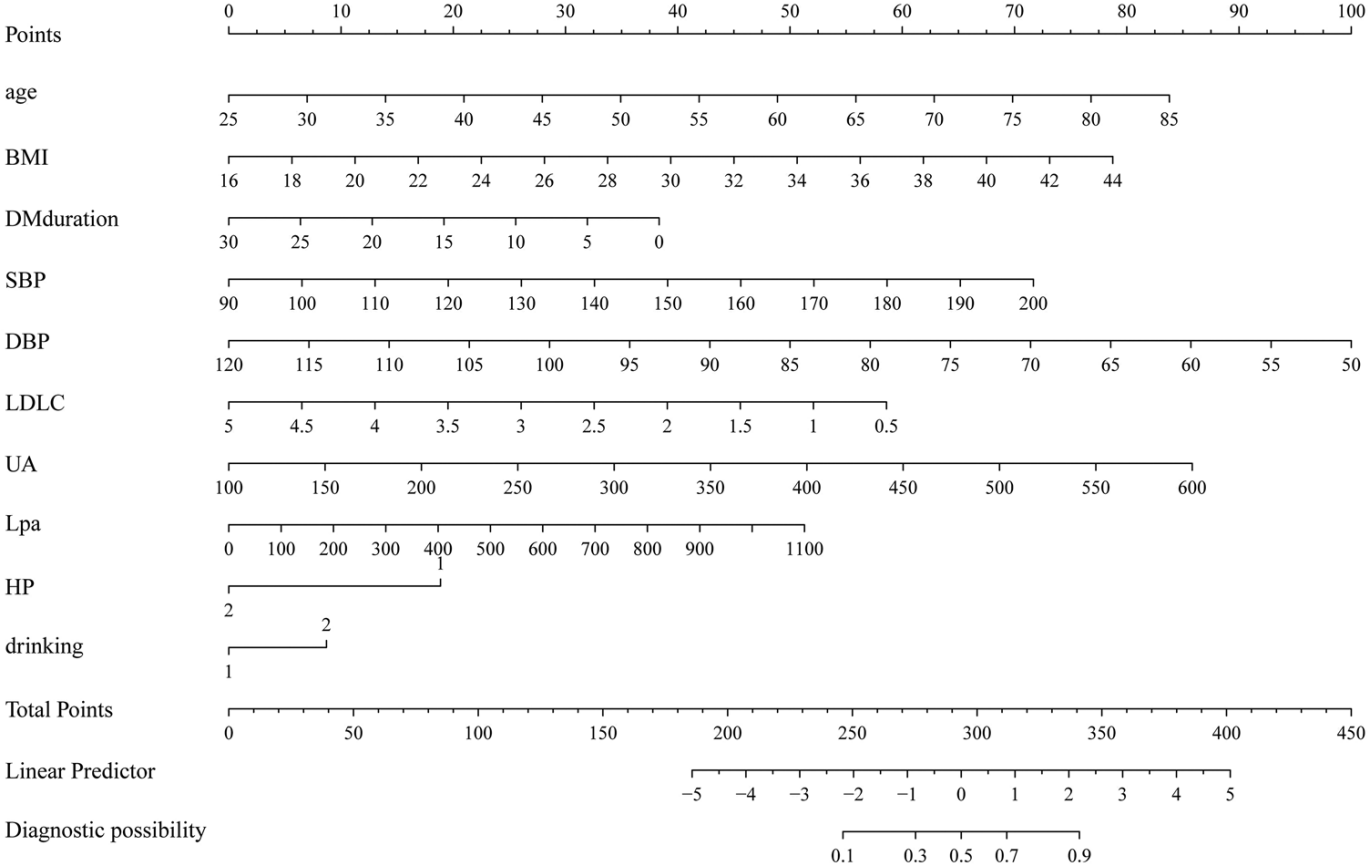
A nomogram for predicting the probability of new-onset ACS in T2DM patients. The nomogram is used by scoring each variable on its corresponding score scale. The scores for all variables are then summed up to obtain the total score, and a vertical line is drawn from the total point row to indicate the estimated probability of new-onset ACS in T2DM patients. BMI, body mass index; SBP, systolic blood pressure; DBP, diastolic blood pressure; LDL-C, low-density lipoprotein cholesterol; UA, uric acid; Lp(a), lipoprotein(a); Hp, hypertension history; ACS, acute coronary syndrome; and T2DM, type 2 diabetes mellitus

The LASSO regression analysis was used to select variables from those shown in Table 1, and the multivariate logistic regression was used to establish the predictive model. The established predictive model included age, BMI, diabetes mellitus duration, SBP, DBP, LDL-C, SUA, Lp(a), hypertension history and alcohol drinking status as predictors. The predictive model was presented using a nomogram that was used to quantitatively predict the risk probability of new-onset ACS in T2DM patients (Fig. 1).

### Weight of predictors

From the nomogram of the predictive model, the single scores of age, BMI, SBP, DBP and SUA were all greater than 60, indicating that these predictors had great weight in the model.

### Predictive model validation

**Fig. 2:**
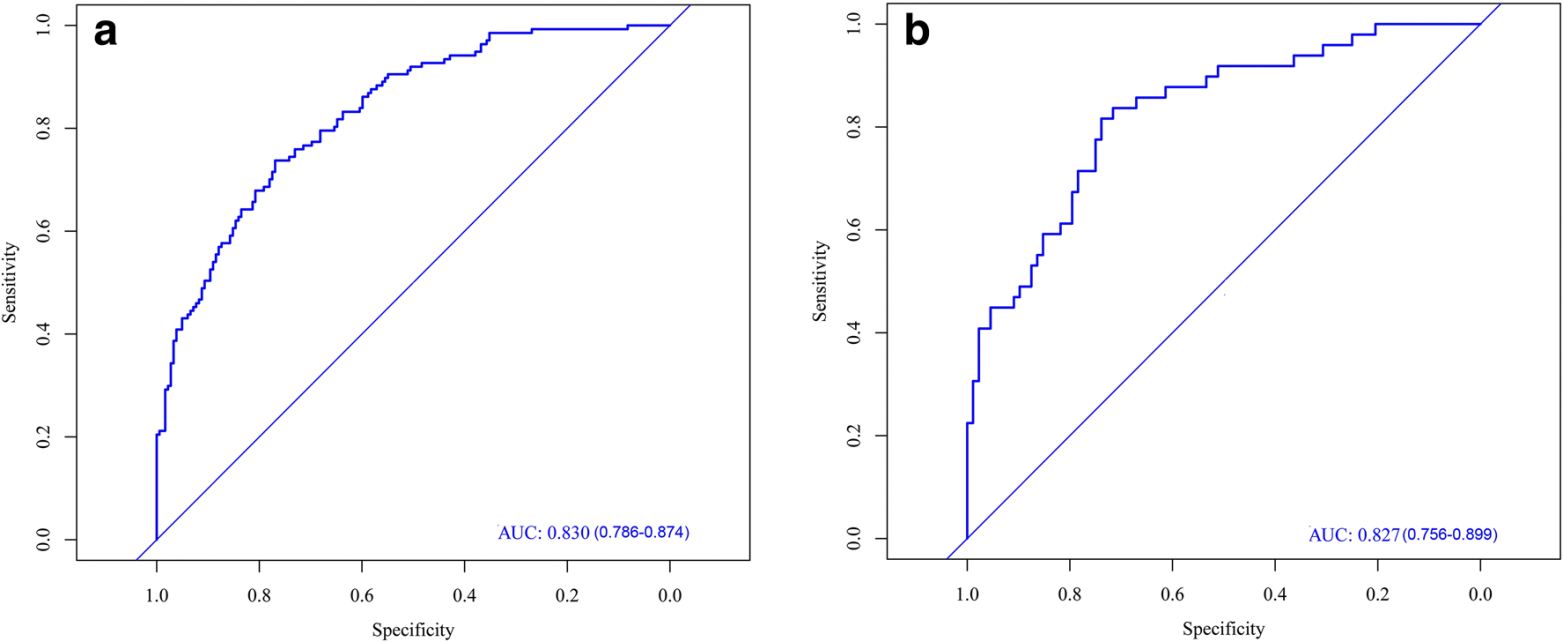
ROC curves **a** from the training set and **b** from the validation set

The AUC was used to evaluate the discriminatory capacity of the predictive model. For the predictive model, the AUC was 0.830 [95% confidence interval (CI) 0.786–0.874], while that of the internal validation set was 0.827 (95% CI 0.756–0.899) (Fig. 2).

**Fig. 3:**
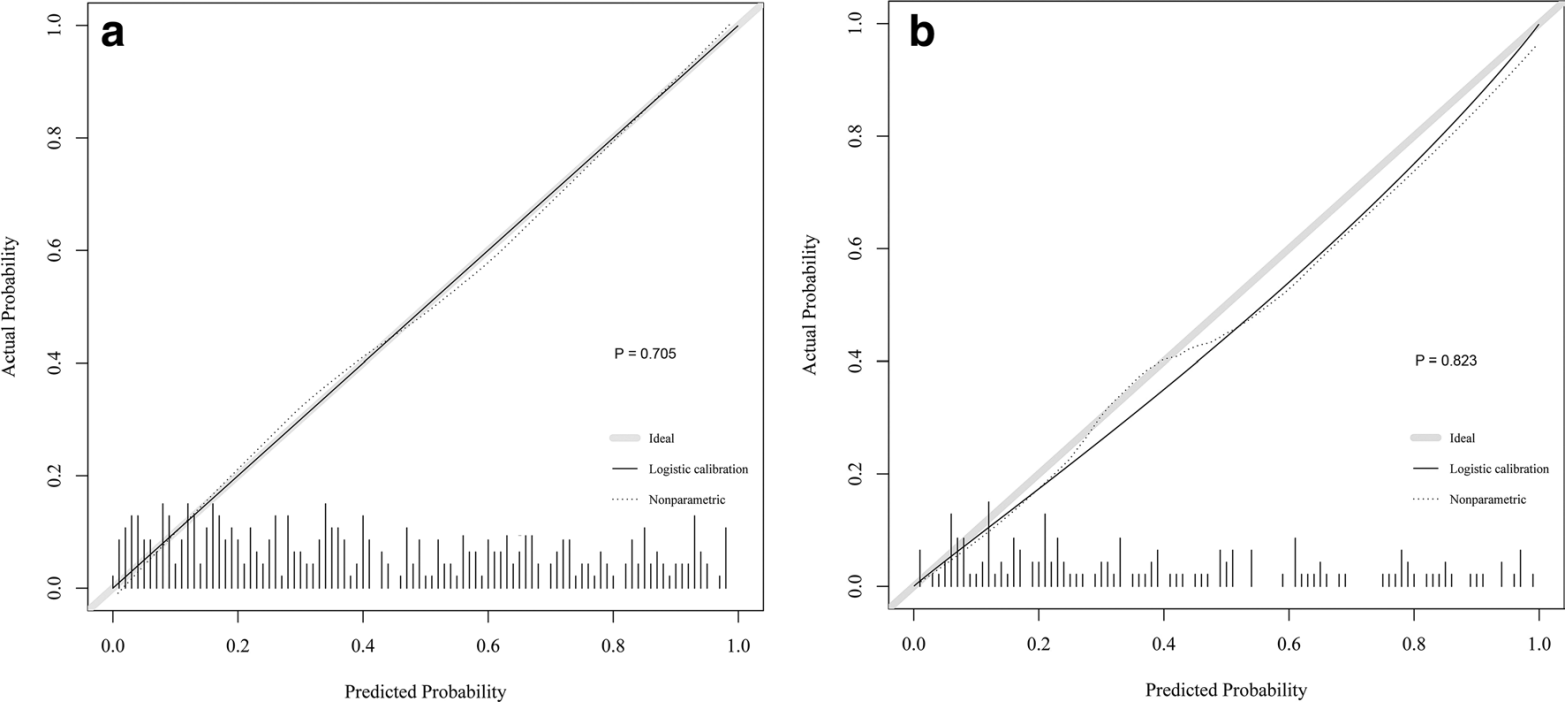
Calibration plots. The shadow line represents a perfect prediction by an ideal model, and the dotted line shows the performance of the training set (**a**) and validation set (**b**). The Hosmer–Lemeshow test yielded a *P* value of 0.705 in the training set (**a**) and 0.823 in the validation set (**b**)

A calibration plot and Hosmer–Lemeshow test were used for the calibration of the predictive model. From the calibration curves, the predictive model and the validation set showed very good fitting degree. As shown by the Hosmer–Lemeshow test, the predicted and actual probability was highly consistent (training set, *P* = 0.705; validation set, *P* = 0.823) (Fig. 3).

**Fig. 4:**
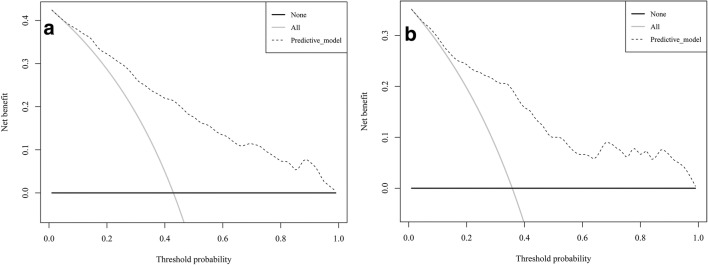
Decision curve analysis. Area within the dotted line, the grey solid line, and the black solid line represents the net benefit. The black solid line indicates that all samples are negative and all were not treated. The grey solid line indicates that all samples were positive and were treated **a** from the training set and **b** from the validation set

DCA was used to evaluate the clinical validity of the predictive model. From the decision curves, the net benefits of the predictive model and the internal validation set were significantly higher than those of the two extreme cases, that is, all people were treated (Fig. 4).

## Discussion

For the first time, we established a risk prediction model with a strong predictive ability and an AUC that reached 0.830. In addition, the predictive model established in this study had high calibration and clinical validity. According to the predictive model, the clinical characteristics of diabetic patients, such as age, BMI, diabetes duration, hypertension history, alcohol drinking status, SBP, DBP, LDL-C, SUA and Lp(a) levels, can be used as predictors. Therefore, in clinical practice, we can predict the risk probability of new-onset ACS in T2DM patients by obtaining information about their general medical history, conducting physical examination and performing serum biochemical indicators testing. The predictive model can guide the formulation of ACS prevention strategies for T2DM in local areas.

Multiple cardiovascular risk factors exist in T2DM patients [9]. Even after adjusting for other factors, fourfold increased risk of heart failure was reported [10]. In 2015, the American Heart Association and the ADA (AHA/ADA) issued a statement [11], indicating that lifestyle, weight management, glycosylated haemoglobin level, hyperglycaemia or hypoglycaemia, hypertension, high cholesterol and hyperlipoproteinaemia are all risk factors for CVD in T2DM patients. At present, the prevalence of T2DM in China continues to rise, and the rate of patients with controlled T2DM is significantly lower than those in developed countries. Furthermore, many patients require PCI for ACS due to diabetes. These patients often show multiple vessel damage and poor prognosis, especially in Northwest China. This situation leads to serious shortage of medical resources. The population in Northwest China is characterized by lower educational and income level than that of Eastern China with a developed economy. The diet is characterized by high salt and oil intakes, and the staple food is mainly carbohydrate. There is an urgent need to establish an economical and effective risk prediction model for new-onset ACS in T2DM patients in this area. This model could provide answers to the factors that are related to the onset of ACS in T2DM patients in this population group; it could also predict the weight of each predictor in the risk prediction model. This will help to develop effective prevention strategies by clinicians in the region.

Previous studies have shown that age [12–16], hypertension [4, 17], obesity [18, 19], hyperuricaemia [20–24], dyslipidaemia [25] are all risk factors for CVD. Although the above results were obtained in the general population, these risk factors remain as predictors in the risk model established in this study. Currently, whether strict blood glucose control can reduce adverse cardiovascular outcomes in diabetic patients is still controversial. In patients with long course of T2DM, strict blood glucose management may not necessarily reduce adverse cardiovascular outcomes [26]. Studies have shown that hyperglycaemia is a relatively weak risk factor for CVD, and the anti-atherosclerosis effect of decreased HbA1c may take more than 10 years to show [27–29]. This present study did not find blood glucose level to be a predictor of ACS in T2DM. However, the risk of ACS in patients with long duration of T2DM was found to be low, but the weight of diabetes duration as a predictor was also low.

Studies have found that moderate drinking can reduce the incidence of cardiovascular events and total mortality in T2DM patients compared to non-drinking. Heavy drinking can increase the risk of cardiovascular events and total mortality in T2DM patients, and the risk and mortality of cardiovascular events in alcoholics showed a dose–response relationship with ethanol consumption [30]. This study found a lower risk of ACS in T2DM patients who drank alcohol than that in non-alcoholic patients, but this study did not quantify alcohol consumption. There is evidence that in hypertensive individuals, aggressive lowering of the blood pressure through antihypertensive therapy may predispose to increased cardiovascular risk due to low DBP [31–33]. This study also found that the lower the DBP in T2DM patients, the higher the risk of ACS.

Elevated Lp(a) and LDL-C are associated with increased risk of CVD [34, 35]. There is an evolving body of evidence supporting the role of Lp(a) in the risk of coronary artery disease, in epidemiological studies [36, 37], and in Mendelian randomization [38] and genome-wide association studies [39]. A recent study demonstrated that LDL-C ≥ 190 mg/dL (≥ 4.91 mmol/L) is associated with an accelerated risk of coronary artery disease [40] and the coexistence of both elevated LDL-C and Lp(a) may exacerbate this risk [41, 42]. This present study found that elevated Lp(a) increased the risk of ACS in T2DM, similar to previous studies in the general population. However, this study found that lower LDL-C levels increased the risk of ACS in T2DM, which is inconsistent with previous studies in the general population. We consider that diabetic patients at high risk of ACS in this region may have had elevated LDL-C in the past and taken statins. Recent studies demonstrated that the relationship between LDL-C and CVD depends on the level of LDL-C and the cumulative exposure time [43, 44].

Finally, our study also demonstrated that age, BMI, SBP, DBP and SUA played a large role in the predictive model. Therefore, in Northwest China, the risk of new-onset ACS in T2DM may be significantly reduced by lowering the body weight, SBP and blood uric acid levels and maintaining an appropriate DBP.

## Limitations

There are some limitations in this study. Firstly, the prediction model established in this study suggests that alcohol drinking can reduce the risk of ACS in T2DM, but there is no detailed quantification of drinking, such as light or large drinking, which may lead to differences in results. Secondly, the prediction model suggests that low LDL-C level may increase the risk of ACS in T2DM. The data of patients in this study did not provide information about the use of lipid-regulating drugs, which might have been used by patients with low LDL-C at admission. Thirdly, although the sample size of this study was small, the AUC of the predictive model and that of the internal validation set were 0.830 (95%CI 0.786–0.874) and 0.827 (95% CI 0.756–0.899), respectively. The predictive model showed very good fitting degree, and the DCA demonstrated a clinically effective predictive model. Finally, due to the large gap in the level of medical care in this region, a single-centre study was conducted to ensure the reliability of the study. Therefore, whether the results of this study can be extended to other regions and countries needs to be further verified by external cohorts.

## Conclusions

In conclusion, we have established a cost-effective risk prediction model in Northwest China, and the application of this model may play an important role in preventing adverse cardiovascular outcomes in T2DM patients in this region.

## Data Availability

The datasets used during the current study are available from the corresponding author on reasonable request.

## Abbreviations

ACS: Acute coronary syndrome
AUC: Area under the curve
BMI: Body mass index
CHD: Coronary heart disease
CI: Confidence interval
CVD: Cardiovascular disease
DBP: Diastolic blood pressure
DCA: Decision curve analysis
LASSO: The least absolute shrinkage and selection operator
LDL-C: Low-density lipoprotein cholesterol
Lp(a): Lipoprotein(a)
OR: Odds ratio
PCIs: Percutaneous coronary interventions
ROC: Receiver operating characteristics
SBP: Systolic blood pressure
SUA: Serum uric acid
T2DM: Type 2 diabetes mellitus
